# Sex-specific but not sexually explicit: pupillary responses to dressed and naked adults

**DOI:** 10.1098/rsos.160963

**Published:** 2017-05-03

**Authors:** Janice Attard-Johnson, Markus Bindemann

**Affiliations:** School of Psychology, University of Kent, Canterbury CT2 7NP, UK

**Keywords:** eye-tracking, pupillary response, viewing behaviour, sexual interest, sexual appeal

## Abstract

Dilation of the pupils is an indicator of an observer's sexual interest in other people, but it remains unresolved whether this response is strengthened or diminished by sexually explicit material. To address this question, this study compared pupillary responses of heterosexual men and women to naked and dressed portraits of male and female adult film actors. Pupillary responses corresponded with observers' self-reported sexual orientation, such that dilation occurred during the viewing of opposite-sex people, but were comparable for naked and dressed targets. These findings indicate that pupillary responses provide a sex-specific measure, but are not sensitive to sexually explicit content.

## 1. Introduction

The presentation of male and female adults elicits dilation in observers' pupils to the people category that matches their sexual interest. This effect is observed with a variety of stimuli, ranging from static images of partially dressed adults [1–3] to photographs of nudes [3–5], and sexually explicit video [6,7]. High sexual explicitness is necessary for eliciting a response pattern that reveals observers' sexual preferences with other physiological approaches, such as phallometric measures [8,9]. This raises ethical concerns and limits the use of such measures in applied and research settings [10]. It remains unclear, however, whether the *level* of sexual exposure also modulates pupillary responses, which level of exposure provides the strongest index of sexual interest with this method, and whether exposure interacts with observer sex.

An early investigation provides evidence that nude images selectively enhance pupillary responses to people of sexual interest [3]. In this study, heterosexual female observers viewed images of two male and two female models presented in various stages of undress. Observers exhibited greatest pupillary dilation to images of naked men in comparison to partially and fully dressed men. However, this effect was only present for some of the male images, and no difference was found for images of women. Moreover, male observers were not included in this study, and pupillary responses were measured crudely with a manual technique.

In a subsequent study, naked images elicited a generalized pupil dilation response in heterosexual men and women that did not differentiate target sex [11]. In contrast to Hamel's [3] findings, this indicates that nudity might also interfere with the measurement of sex preference effects. However, more recent investigations with more precise eye-tracking equipment also indicate that the pupillary responses of heterosexual male observers to nude [6,7] and partially nude people [1,2] reflect their sexual interests. By contrast, the pupils of heterosexual female observers dilated indiscriminately for both sexes [1,6], or more to same-sex stimuli [7]. However, these studies did not directly compare responses to nude and partially nude stimuli with images of dressed persons and therefore cannot address whether these image types provide different indexes of sexual interest.

To provide a more direct comparison, a recent investigation contrasted observers' pupillary responses to video footage of nude persons performing sexual acts with footage of dressed persons discussing the weather [12]. In this study, pupil dilation patterns were stronger for sexually explicit stimuli, and these materials also yielded clearer sex differences. However, the sexually explicit and non-explicit stimuli were not systematically matched for person identity and scene content in this study, which raises the possibility that these factors contributed to the difference in pupillary response patterns. Consequently, these findings cannot reveal fully whether more explicit sexual information yields stronger pupil dilation patterns linked to sexual orientation.

To systematically investigate how level of nudity affects pupillary responses, the current study employed highly controlled portraits of dressed and naked adults, which were matched for identity, pose and image content. An intermediate stage of nudity was also presented, by blurring genital and chest areas of the naked stimuli. Observers' fixations on these images were analysed briefly to confirm that they were looking at the person content. We then analysed pupillary responses and correlated these with observers' sexual appeal ratings of the depicted adults.

## 2. Method

### 2.1. Participants

Fifty-two (28 females, 24 males) students from the University of Kent, with a mean age of 22.4 years (s.d. = 5.7), participated in this study. Only participants who reported to be exclusively or predominantly heterosexual, by recording ‘0’ or ‘1’ on the 7-point Kinsey Scale [13,14] in an online pre-screen, were invited to take part.

To confirm sexual orientation on taking part in the experiment, participants again completed the Kinsey scale. The Modified Klein Sexual Orientation Grid (MKSOG) was also administered as a more detailed measure of sexual orientation [15]. On this scale, participants report sexual attractions (‘to whom are you sexually attracted?’) and fantasies (‘about whom do you have sexual fantasies?’) for the past, present and ideal future on 7-point Likert scales, which range from ‘other sex only’ to ‘same sex only’.

Of 24 male observers, 23 reported to be ‘completely heterosexual’ and one selected ‘predominantly heterosexual’ (corresponding to ‘0’ and ‘1’, respectively) on the Kinsey scale. Of 28 female observers, 17 reported to be ‘completely heterosexual’ and 11 selected ‘predominantly heterosexual’ on this scale. These responses were confirmed with the MKSOG. Responses for sexual attraction and fantasies were combined and revealed means of 1.2 (s.d. = 0.2) and 1.6 (s.d. = 0.4) for male and female participants, indicating a strong sexual preference for the opposite sex. Participants with a score that was three standard deviations above these means were excluded from further analysis. A further female participant that produced pupillary responses that were three standard deviations above the mean pupillary responses was excluded. This resulted in the exclusion of experimental data for four male and three female observers.

### 2.2. Stimuli

Photographs of six men and six women were selected from ‘XXX 30 Porn-Star Portraits’ [16]. Each of these targets was portrayed dressed and naked in matching poses on a plain background, which measured 600 by 768 pixels at a resolution of 72 ppi. To create an intermediate nudity condition, the pelvic region of the naked male targets and the breast and pelvic region of the naked female targets were blurred using a graphics software (Adobe Photoshop CS3, Gaussian Blur with 340 pixel radius). This resulted in a total of 36 photographs, comprising 12 images for each of three exposure conditions (dressed, blurred and naked).

**Figure 1.**
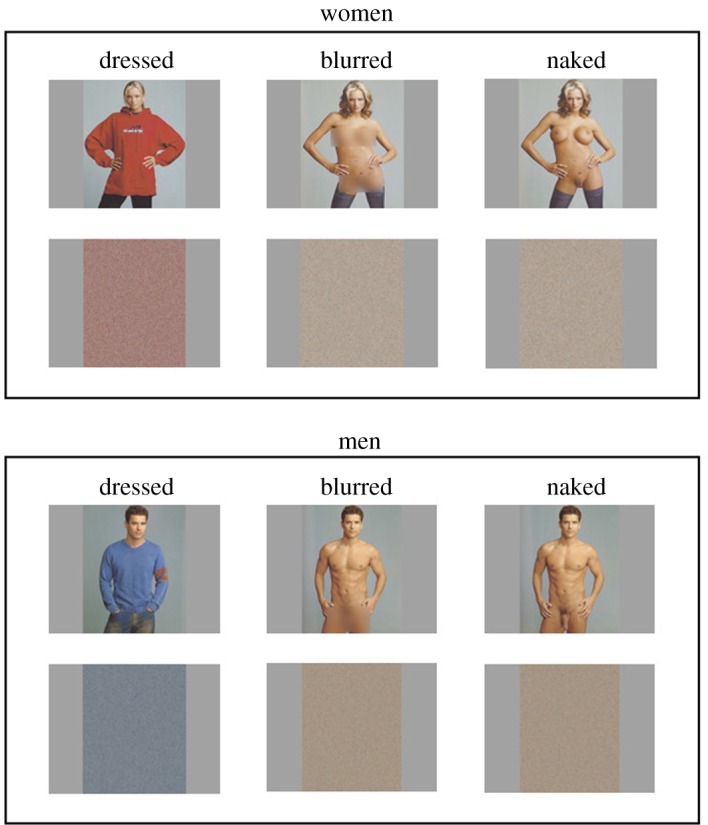
Example stimuli of dressed, blurred and naked women and men, and the corresponding control images. Naked and dressed photographs are from *XXX 30 Porn-Star Portraits* by Timothy Greenfield-Sanders. Copyright © 2004 by Timothy Greenfield-Sanders. Used with permission of Bulfinch/Hachette Book Group USA, Inc. All rights reserved.

Control stimuli were also created to assess the potential effect of low-level stimulus properties on pupillary responses by randomizing the pixels in each photograph (for an illustration, see figure 1). The content of the resulting images is no longer recognizable but colour and mean image luminance are retained (for similar approaches, see [1,17]).

### 2.3. Eye-tracking

Eye movements and pupillary responses were recorded with an SR-Research Eyelink 1000 eye tracker, running at 1000 Hz sampling rate, a spatial resolution of less than 0.01° of visual angle, a gaze position accuracy of less than 0.5°, and a pupil size resolution of 0.1% of area. The Eyelink 1000 measures corneal reflection and dark pupil with a video-based infrared camera, and computes the number of pixels that are occluded by participants' pupils. In this system, a measurement of pupil diameter is recorded at every fixation point as an integer that ranges from 400 to 16 000 units. The stimuli were displayed on a 21“ colour monitor, with a screen resolution of 1024 × 768 pixels. Viewing was binocular but only participants' left eye was tracked. A chin rest was applied to minimize head movements and maintain a viewing distance of 60 cm from the display monitor.

### 2.4. Procedure

Participants were invited to take part in an experiment on sexual interest that involved viewing images of dressed and naked men and women, but were kept naive to the full purpose until the end. Subjects were seated in a quiet windowless room with consistent artificial lighting. The participants' left eye was tracked and calibrated using the standard Eyelink procedure. Thus, participants fixated a series of nine target points on the display monitor. Fixation accuracy was then validated against a second series of nine targets. Calibration was repeated if poor measurement accuracy (less than 0.5°) was indicated.

Participants were instructed to rate the personal sexual appeal of all 72 images. Each trial started with a drift correction, which required fixation of a central target point, followed by a grey screen for 1000 milliseconds, and the target stimulus. Participants recorded their responses on a standard keyboard using a 7-point scale ranging from 1 (‘not at all sexually appealing’) to 7 (‘extremely sexually appealing’). Participants were instructed to keep their fingers on these keys at all times. Once a response was recorded, the target was replaced with a grey screen for 1000 milliseconds, after which the next trial began. The intact scenes and control images were randomly intermixed for each participant by the Eyelink software and interspersed by a short break every 24 trials. On completion of the eye-tracking task, participants completed the Kinsey scale and MKSOG to confirm sexual orientation (see Participants section).

## 3. Results

### 3.1. Sexual appeal ratings

**Figure 2.**
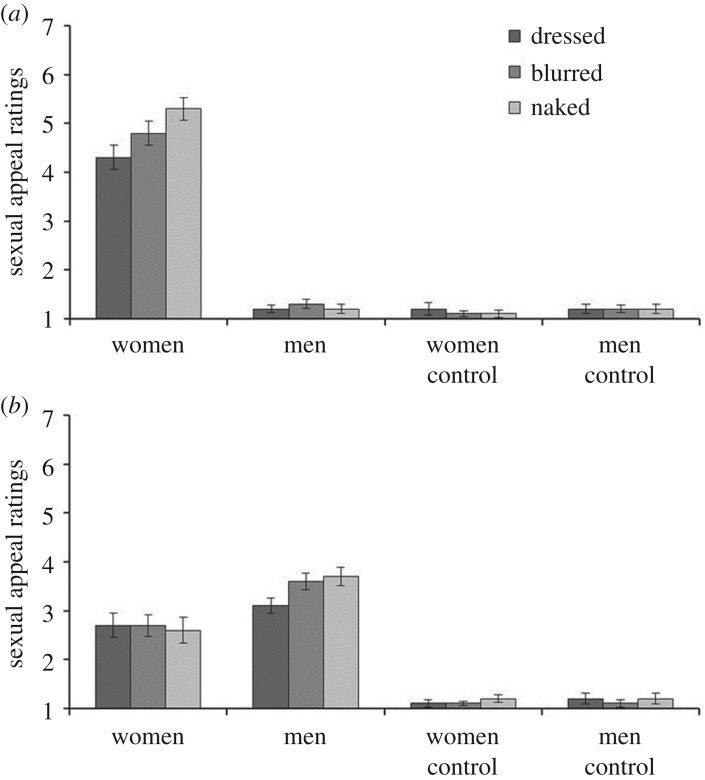
Sexual appeal ratings for male (*a*) and female (*b*) observers. Error bars represent the standard error of the means.

The mean sexual appeal ratings for each stimulus sex (men, women) and exposure condition (dressed, blurred, naked) are illustrated in figure 2 for male and female observers. A 2 (observer sex) × 2 (stimulus sex) × 3 (exposure condition) mixed-factor ANOVA revealed a three-way interaction, *F*_2,86_ = 20.82, *p* < 0.001, partial *η*^2^ = 0.33. To analyse this interaction, separate 2 (stimulus sex) × 3 (exposure condition) within-subjects ANOVAs were performed for male and female observers.

For male observers, this revealed an interaction between stimulus sex and exposure condition, *F*_2,38_ = 15.27, *p* < 0.001, partial *η*^2^ = 0.45. Bonferroni-corrected pairwise comparisons show that male observers rated female targets as more sexually appealing than male targets in all exposure conditions, all *p*s < 0.001. In addition, men rated naked women as more sexually appealing than blurred and dressed women, both *p*s ≤ 0.01, and blurred women as more sexually appealing than dressed women, *p* < 0.001.

The equivalent analysis also revealed an interaction of stimulus sex and exposure condition in female observers, *F*_2,48_ = 7.84, *p* < 0.01, partial *η*^2^ = 0.25. Bonferroni-corrected pairwise comparisons showed that female observers rated men as more sexually appealing than women in the blurred and naked conditions, both *p*s < 0.001, but not in the dressed condition, *p* = 0.15. Furthermore, naked and blurred men were rated as more sexually appealing than dressed men, both *p*s < 0.01, but did not differ from each other, *p* > 0.25.

Finally, a separate 2 (observer sex) × 2 (stimulus sex) × 3 (exposure condition) mixed-factor ANOVA was conducted on the sexual appeal ratings for the control images. This did not reveal main effects or interactions, all *F*s ≤ 2.62, *p*s ≥ 0.08, partial *η*^2^s ≤ 0.06.

### 3.2. Response times

**Figure 3.**
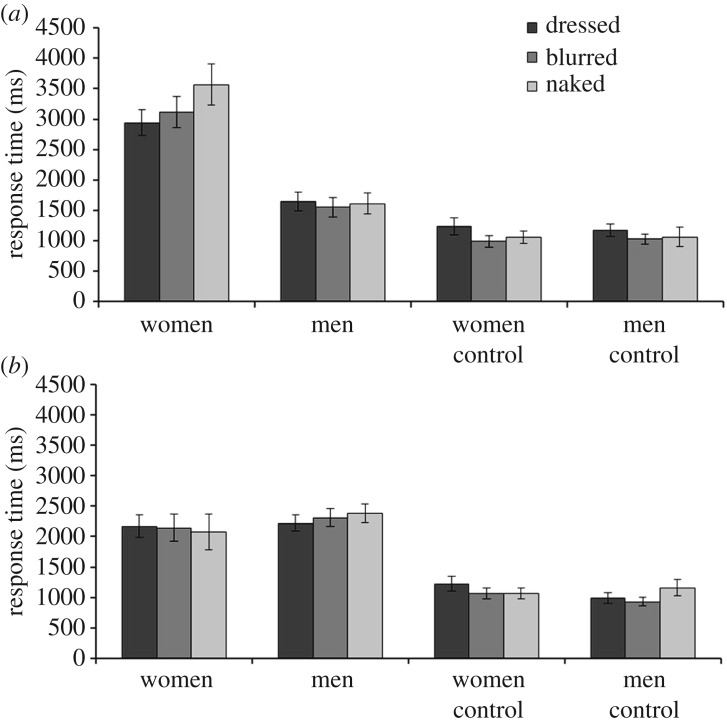
Mean response times (ms) for all stimulus categories for male (*a*) and female (*b*) observers. Error bars represent standard errors of the means.

Response times for sexual appeal ratings were also analysed and are illustrated in figure 3. A 2 (observer sex) × 2 (stimulus sex) × 3 (exposure condition) mixed-factor ANOVA of this data revealed a three-way interaction, *F*_2,86_ = 4.42, *p* < 0.05, partial *η*^2^ = 0.09. Therefore, separate 2 (stimulus sex) × 3 (exposure condition) within-subjects ANOVAs were performed for male and female observers.

For male observers, a main effect of stimulus sex was found, *F*_1,19_ = 21.58, *p* < 0.001, partial *η*^2^ = 0.53, with faster response times for images of men than women. A main effect of exposure condition, *F*_2,38_ = 1.59, *p* = 0.22, partial *η*^2^ = 0.08, and an interaction between factors were not present, *F*_2,38_ = 2.22, *p* = 0.12, partial *η*^2^ = 0.11. For female observers, main effects of stimulus sex, *F*_1,24_ = 2.23, *p* = 0.15, partial *η*^2^ = 0.09, exposure condition, *F*_2,48_ = 0.13, *p* = 0.88, partial *η*^2^ = 0.01, and an interaction were not found, *F*_2,48_ = 2.50, *p* = 0.09, partial *η*^2^ = 0.09.

A 2 (observer sex) × 2 (stimulus sex) × 3 (exposure condition) mixed-factor ANOVA for the control images revealed a main effect of exposure condition, *F*_2,86_ = 4.44, *p* < 0.05, partial *η*^2^ = 0.09, whereby the control images for the dressed condition produced slower responses compared to the blurred condition, *p* < 0.001. No other differences were found, all *F*s ≤ 1.89, *p*s ≥ 0.16, partial *η*^2^s ≤ 0.04.

### 3.3. Eye-fixation check

**Figure 4.**
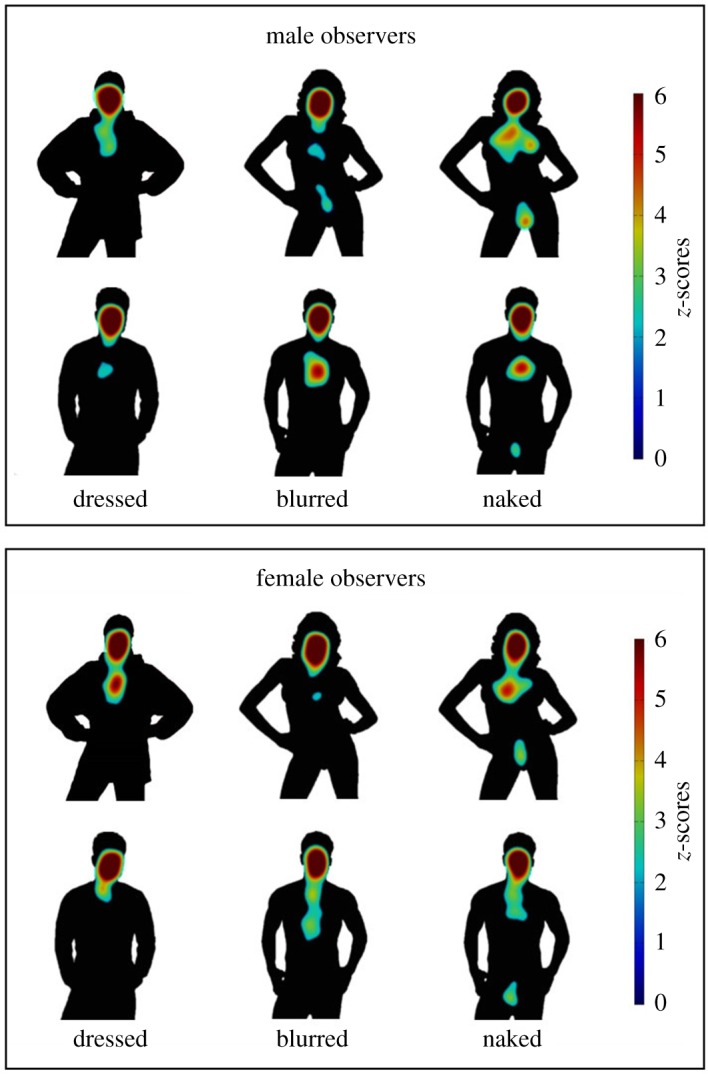
Distribution of fixations for a female (top row in panel) and a male (bottom row in panel) target in the dressed, blurred and naked exposure conditions for male and female observers.

The fixation locations were analysed to confirm that observers looked at the person content of the stimuli, and the sexually relevant regions of the nude images. All eye movements were pre-processed by merging fixations of less than 80 ms with the preceding or following fixation if that fell within half a degree of visual angle (for similar approaches, see, e.g. [1,18]). Blinks and fixations outside the display monitor were excluded. To provide a brief overview of viewing behaviour, the fixations of each trial were then fitted with a Gaussian (radius = 3° of visual angle), and a *z*-scored distribution of these Gaussians was plotted (for similar analysis, see, e.g. [19,20]). Figure 4 shows these fixation maps superimposed on silhouettes of example images from each person-category for male and female observers. These data demonstrate that observers fixated the targets' faces in all conditions but increased attention to the chest and pelvis in the naked conditions (a detailed analysis of the percentage fixations to the head, chest and pelvis is reported in the electronic supplementary materials available online).

### 3.4. Pupil dilation: comparison of conditions

Pupillary responses were then computed by calculating the mean pupil size for all fixations across the duration of the stimulus displays. These values were used to compute an overall mean, across all stimuli, for each participant. These pupillary responses were evaluated for outliers, which resulted in the exclusion of one female participant with a score three standard deviations above the group mean. The percentage difference (i.e. an increase or decrease) in pupil size from the overall mean was then computed for all conditions, using the formula: 100 − (mean pupil size for condition × 100/overall pupil mean). For the resulting scores, a value of zero indicates no change in pupil size and positive or negative scores reflect relatively larger (dilation) or smaller (constriction) pupil sizes for a stimulus category (for similar approaches, see [1,21,22].

**Figure 5.**
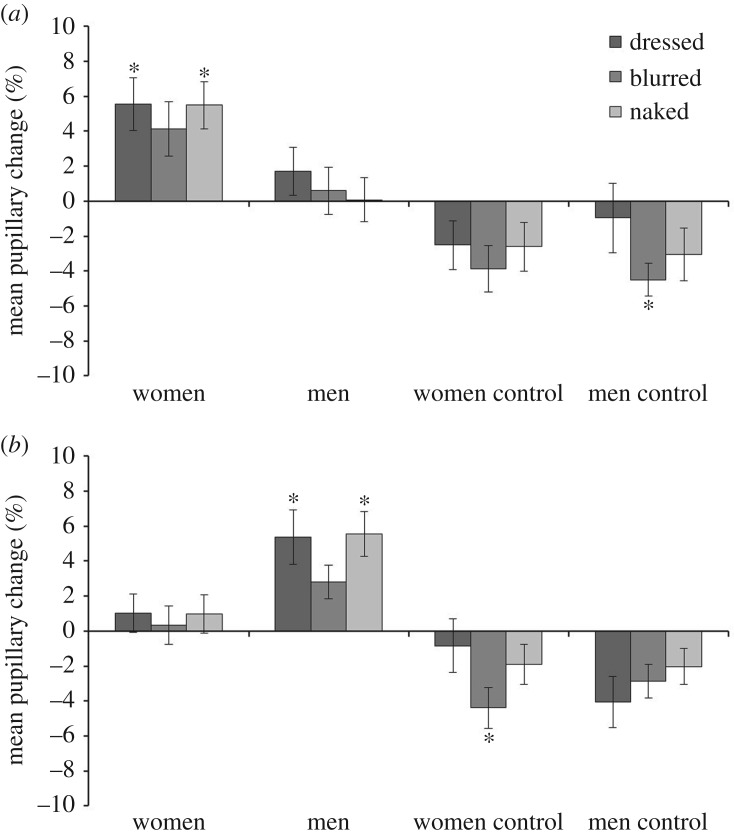
Percentage pupillary change for all stimulus categories for male (*a*) and female (*b*) observers. Error bars represent standard error of the means. *Note:* * represents *p* < 0.004 in the one-sample *t*-tests (*alpha* corrected for multiple comparisons).

These pupillary responses were compared for male and female observers across all conditions (see figure 5). A 2 (observer sex) × 2 (stimulus sex) × 3 (exposure condition) mixed-factor ANOVA of this data revealed an interaction of stimulus sex and observer sex, *F*_1,43_ = 21.72, *p* < 0.001, partial *η*^2^ = 0.34. Bonferroni-corrected pairwise comparisons showed that male observers' pupils were larger while viewing women than men, *p* < 0.01, whereas female observers displayed the opposite effect, *p* < 0.01. No other main effects or interactions were found, all *F*s ≤ 1.01, *p*s ≥ 0.37, partial *η*^2^s ≤ 0.02. In addition, a 2 (observer sex) × 2 (stimulus sex) × 3 (exposure condition) mixed-factor ANOVA was also conducted for the control images (see figure 5). This revealed no main effects or interactions, all *F*s ≤ 2.11, *p*s ≥ 0.13, partial *η*^2^s ≤ 0.05.

To determine whether this pattern could be accounted for by differences in response times to the experimental conditions, one-way ANCOVAs were conducted on the pupillary data for images of men and women with response time as the covariate. For images of men, response time did not predict pupil size change, *F*_1,42_ = 0.11, *p* = 0.74, partial *η*^2^ = 0.00. And when response time was adjusted for, pupil size was still reliably larger for these images in female observers compared to males, *F*_1,42_ = 8.11, *p* < 0.01, partial *η*^2^ = 0.16. The equivalent analysis for images of women showed that response time relates to some of the difference between these observer groups, *F*_1,42_ = 7.14, *p* = 0.01, partial *η*^2^s = 0.15. However, a marginally significant effect suggests that pupil size was still larger for male than female observers when response times was adjusted for, *F*_1,42_ = 3.86, *p* = 0.06, partial *η*^2^s = 0.08. These analyses therefore suggest that any differences in viewing time across experimental conditions cannot explain the pupil dilation patterns.

### 3.5. Pupil dilation: comparison to baseline

Pupillary responses were also compared with a baseline that reflects the mean pupil size during the viewing of all stimuli via a series of one-sample *t*-tests (with *alpha* corrected at *p* < 0.004 for multiple comparisons). The pupils of male observers were larger than baseline during the viewing of dressed, and naked women, *t*_19_ = 3.64, *p* < 0.004, *d* = 1.67, and *t*_19_ = 4.07, *p* < 0.004, *d* = 1.87, respectively. A similar trend was observed for blurred women, but this did not reach significance, *t*_19_ = 2.72, *p* = 0.014, *d* = 1.25. In contrast, pupil size did not differ from baseline for dressed, *t*_19_ = 1.25, *p* = 0.23, *d* = 0.57, blurred, *t*_19_ = 0.52, *p* = 0.61, *d* = 0.24, and naked men, *t*_19_ = 0.04, *p* = 0.97, *d* = 0.02. Pupillary responses to control scenes were consistently below baseline but these differences were not reliable, all *t*s ≤ 3.05, *p*s ≥ 0.007, *d*s ≤ 1.40, except for blurred men, *t*_19_ = 4.73, *p* < 0.004, *d* = 2.17.

In female observers, dressed and naked men elicited pupil sizes above baseline, *t*_24_ = 3.45, *p* < 0.004, *d* = 1.41 and *t*_24_ = 4.32, *p* < 0.001, *d* = 1.76, respectively. Blurred men produced a similar but non-significant effect, *t*_24_ = 2.98, *p* = 0.006, *d* = 1.22. By contrast, pupil sizes did not differ reliably from baseline for dressed, *t*_24_ = 0.93, *p* = 0.36, *d* = 0.38, blurred, *t*_24_ = 0.33, *p* = 0.75, *d* = 0.13, and naked women, *t*_24_ = 0.95, *p* = 0.35, *d* = 0.39. Finally, pupil sizes for the control images were consistently smaller than baseline across conditions but these effects were not significant, all *t*s ≤ 2.93, *p*s ≥ 0.007, *d*s ≤ 1.20, except for blurred women, *t*_24_ = 3.75, *p* < 0.004, *d* = 1.53.

### 3.6. Correlation of sexual appeal and pupillary responses

Sexual appeal ratings were also correlated with mean pupillary change. For this analysis, the control conditions were excluded and the data for male and female targets was combined. The distribution of sexual appeal ratings was skewed. Therefore, non-parametric Spearman's correlations are reported. For male observers, positive correlations between pupillary change and sexual appeal ratings were found for dressed, *r_s_*(38) = 0.33, *p* < 0.05, and naked stimuli, *r_s_*(38) = 0.49, *p* < 0.01, but not for blurred stimuli, *r_s_*(38) = 0.26, *p* = 0.11. For female observers, a correlation was not found for dressed, *r_s_*(48) = 0.19, *p* = 0.18, and naked stimuli, *r_s_*(48) = 0.18, *p* = 0.20, but was present for blurred person photographs, *r_s_*(48) = 0.28 *p* = 0.05.

## 4. Discussion

This study examined whether pupillary responses to the visual presentation of men and women are influenced by different levels of sexual exposure. More specifically, we sought to determine whether one of these conditions (dressed, partially naked or naked) provides a clearer index of sexual interest, and whether this interacts with observer sex. This experiment showed pupillary responses that were consistent with observer's self-reported sexual preferences. Thus, pictures of women elicited a clear pupillary dilation in heterosexual male observers that was not present when viewing men or control images. In contrast, pupil size was largest in heterosexual female observers during the viewing of men compared to women and control images.

When pupillary responses were broken down by exposure condition, strong dilation patterns for both dressed and naked persons emerged. Only a small set of studies have directly compared pupillary responses to such images, with inconsistent results. One study assessed pupillary responses of heterosexual female observers and found enhanced dilation for naked male images [3]. However, a later study revealed a generalized dilation response for naked stimuli of both sexes in heterosexual males and females [11].

Several factors could account for these discrepancies. For example, such a discrepancy in findings might reflect the use of different eye-tracking methods for measuring pupil size, which range from elementary pupillometry systems that record pupil diameter only every minute [3] or every 0.5 s [11], to state-of-the-art equipment with millisecond precision [12]. Furthermore, it is unclear whether these studies controlled for stimulus factors such as identity, colour and pose. Aboyoun & Dabbs [11], for example, intermixed images of Caucasian and African American men and women, and different identities were presented in the naked and dressed conditions. Strong differences in colour tone arising from such a mixture of identities and race could have interfered with pupillary responses to the sexual content of these images [23,24]. Similarly, Watts *et al*. [12] compared responses to people in pornographic footage with recordings of other people discussing weather, leaving open the possibility that their results might reflect differences in person identity or non-person content. The current study improves on these previous attempts by using sophisticated contemporary eye-tracking technology in combination with highly controlled stimuli. Under these conditions, pupillary responses to images of men and women appear to be sex-specific but not sensitive to the sexual explicitness of the materials.

Naked images of people have been shown to elicit a stronger recording of arousal than dressed images when this is measured with other physiological measures, such as genital response and skin conductance [8,25–27]. It is unclear why a similar pattern is not found with pupillary responses here. Pupil dilation is an instantaneous response [28], so it is possible that a change in pupil size is elicited with lower levels of sexual arousal than is necessary for other physiological measures. As such, images of dressed people may provide sufficient arousal for eliciting a similarly strong dilation response to naked images under the current conditions.

The responses of male observers to stimuli depicting women converge with previous research, which has also shown increases in pupil size to such content [1,2,5–7]. In the current study, female observers also showed stronger dilation for photographs depicting the opposite sex. In the sex literature, there is mixed evidence with regard to the response patterns of heterosexual women. Some studies have revealed pupil dilation in female observers that is indistinguishable to sexual content of men and women [1,6,7] or stronger for the opposite sex [3,4,12,22]. In light of these differences, the current study also investigated whether nudity influences the pupillary responses of *heterosexual females* by enhancing [3] or diminishing any sexual preference effects [11]. In this experiment, these observers recorded clear dilation patterns for images depicting persons of the opposite sex, consistent with their sexual orientation. More importantly, this pattern was present for naked *and* dressed images. This suggests that image nudity cannot explain the inconsistent dilation patterns that have been recorded across studies in heterosexual females. Instead, it is possible that such inconsistencies arise from internal factors, such as fluctuations in the hormonal cycle and affective state, that may affect women more than men (for a review, see [29]).

As with previous studies, these pupil responses also correlated for male observers with the sexual appeal ratings that were provided for these photographs, which indicates a direct link between sexual interest and pupil size [1,6,7]. In male observers, this was found for the naked and dressed image conditions, but not for blurred stimuli. This could be due to the weaker pupillary responses to these images, and is discussed in more detail below. In line with previous research, these correlations were weaker or not present in female observers whose responses only correlated for the blurred condition [6,7]. In these observers, the differences in sexual appeal ratings of male and female targets were smaller than those obtained for male observers. This could therefore account for the lack of further reliable correlations between sexual appeal ratings and pupil size in female observers.

This experiment also included a third condition in which the sexual regions of the targets were blurred to provide a partially naked condition. Pupillary responses to these blurred images also showed dilation for the preferred target sex, but this effect was weaker in comparison to the dressed and naked stimuli. It is unclear why this is the case. However, one possible explanation could be that the blurred image regions interfered with pupillary responses. When a viewer's eye is directed from a distant to a nearby object, the image becomes ‘out-of-focus’. Consequently, an accommodation response is triggered, whereby the pupils constrict to increase depth of focus and improve image quality [30]. It is possible that a similar reflex occurred here, whereby the partially blurred images were processed as ‘out-of-focus’ stimuli, triggering the accommodation reflex and pupil constriction. This constriction may have counteracted pupil dilation that was elicited by the sexual interest of the blurred stimuli.

## Supplementary Material

Fixation Data

## Data accessibility

Full analysis for viewing behaviour in this study is included in the electronic supplementary material. The data underlying the analyses for viewing behaviour, pupillary responses and sexual appeal ratings can be found in the Dryad Digital Repository, http://dx.doi.org/10.5061/dryad.22242 [31].

## References

[1] Attard-Johnson J, Bindemann M, Ó Ciardha C 2016 Pupillary response as an age-specific measure of sexual interest: an exploratory study. Arch. Sex. Behav. 45, 855–870. (doi:10.1007/s10508-015-0681-3) 26857377 10.1007/s10508-015-0681-3 PMC4820473

[2] Attard-Johnson J, Bindemann M, Ó Ciardha C In press Heterosexual, homosexual, and bisexual men's pupillary responses to persons at different stages of sexual development. J. Sex Res. (doi:10.1080/00224499.2016.1241857) 10.1080/00224499.2016.1241857 27925771

[3] Hamel RF 1974 Female subjective and pupillary reaction to nude male and female figures. J. Psychol. 87, 171–175. (doi:10.1080/00223980.1974.9915687) 4443952 10.1080/00223980.1974.9915687

[4] Hess EH, Polt JM 1960 Pupil size as related to interest value of visual stimuli. Science 132, 349–350. (doi:10.1126/science.132.3423.349) 14401489 10.1126/science.132.3423.349

[5] Hess EH, Seltzer AL, Shlien JM 1965 Pupil response of hetero- and homosexual males to pictures of men and women: a pilot study. J. Abnorm. Psychol. 70, 165–168. (doi:10.1037/h0021978) 14297654 10.1037/h0021978

[6] Rieger G, Cash BM, Merrill SM, Jones-Rounds J, Dharmavaram SM, Savin-Williams RC 2015 Sexual arousal: the correspondence of eyes and genitals. Biol. Psychol. 104, 56–64. (doi:10.1016/j.biopsycho.2014.11.009) 25603717 10.1016/j.biopsycho.2014.11.009

[7] Rieger G, Savin-Williams RC 2012 The eyes have it: sex and sexual orientation differences in pupil dilation patterns. PLoS ONE 7, e40256 (doi:10.137/journal.pone.0040256) 22870196 10.1371/journal.pone.0040256 PMC3411709

[8] Abel GG, Blanchard EB, Barlow DH 1981 Measurement of sexual arousal in several paraphilias: the effects of stimulus modality, instructional set and stimulus content on the objective. Behav. Res. Ther. 19, 25–33. (doi:10.1016/0005-7967(81)90109-1) 7225034 10.1016/0005-7967(81)90109-1

[9] Barker JG, Howell RJ 1992 The plethysmography: a review of recent literature. Bull. Am. Acad. Psychiatry Law 20, 13–25. 1576372

[10] Merdian HL, Jones DT 2011 Phallometric assessment of sexual arousal. In International perspectives on the assessment and treatment of sexual offenders: theory, practice, and research (eds Boer DP, Eher R, Craig LA, Miner MH, Pfäfflin F), pp. 141–170. Chichester, UK: Wiley.

[11] Aboyoun DC, Dabbs JM 1998 The Hess pupil dilation findings: sex or novelty*.* Soc. Behav. Pers. 26, 415–419. (doi:10.2224/sbp.1998.26.4.415)

[12] Watts TM, Holmes L, Savin-Williams RC, Rieger G 2017 Pupil dilation to explicit and non-explicit sexual stimuli. Arch. Sex. Behav. 46, 155–165. (doi:10.1007/s10508-016-0801-8) 27527877 10.1007/s10508-016-0801-8

[13] Kinsey AC, Pomeroy WB, Martin CE, Gebhard PH 1953 Sexual behavior in the human female. Bloomington, IN: Indiana University Press.

[14] Kinsey AC, Pomeroy WB, Martin CE 1948 Sexual behavior in the human male. Bloomington, IN: Indiana University Press.

[15] Klein F, Sepekoff B, Wolf TI 1985 Sexual orientation: a multi-variable dynamic process. J. Homosex. 11, 35–49. (doi:10.1300/J082v11n01_04) 10.1300/J082v11n01_04 4056393

[16] Greenfield-Sanders T 2004 XXX 30 porn-star portraits. New York, NY: Bulfinch Press.

[17] Henderson RR, Bradley MM, Lang PJ 2014 Modulation of the initial light reflex during affective picture viewing. Psychophysiology 51, 815–818. (doi:10.1111/psyp.12236) 24849784 10.1111/psyp.12236 PMC4329731

[18] Attard J, Bindemann M 2013 Establishing the duration of crimes: an individual differences and eye tracking investigation into time estimation. Appl. Cogn. Psychol. 28, 215–225. (doi:10.1002/acp.2983)

[19] Bindemann M, Scheepers C, Ferguson HJ, Burton AM 2010 Face, body, and centre of gravity mediate person detection in natural scenes. J. Exp. Psychol. Hum. Percept. Perform. 36, 1477–1485. (doi:10.1037/a0019057) 20695695 10.1037/a0019057

[20] Blais C, Jack RE, Scheepers C, Fiset D, Caldara R 2008 Culture shapes how we look at faces. PLoS ONE 3, e3022 (doi:10.1371/journal.pone.0003022) 18714387 10.1371/journal.pone.0003022 PMC2515341

[21] Dabbs JM Jr 1997 Testosterone and pupillary response to auditory sexual stimuli. Physiol. Behav. 62, 909–912. (doi:10.1016/S0031-9384(97)00268-0) 9284516 10.1016/s0031-9384(97)00268-0

[22] Laeng B, Falkenberg L 2007 Women's pupillary responses to sexually significant others during the hormonal cycle. Horm. Behav. 52, 520–530. (doi:10.1016/j.yhbeh.2007.07.013) 17870074 10.1016/j.yhbeh.2007.07.013

[23] Kohn M, Clynes M 1996 Color dynamics of the pupil. Ann. NY. Acad. Sci. 156, 931–950. (doi:10.1111/j.1749-6632.1969.tb14024.x) 10.1111/j.1749-6632.1969.tb14024.x 5258025

[24] Lobato-Rincón LL, del Cabanillas-Campos MC, Bonnin-Arias C, Chamorro-Gutierrez E, Murciano-Cespedosa A, Sanchez-Ramos Roda C 2014 Pupillary behavior in relation to wavelength and age. Front. Hum. Neurosci. 8, 1–8. (doi:10.3389/fnhum.2014.00221) 24795595 10.3389/fnhum.2014.00221 PMC4001033

[25] Kuban M, Barbaree HE, Blanchard R 1999 A comparison of volume and circumference phallometry: response magnitude and method agreement. Arch. Sex. Behav. 28, 345–359. (doi:10.1023/A:1018700813140) 10553495 10.1023/a:1018700813140

[26] Malcolm PB, Andrews DA, Quinsey VL 1993 Discriminant and predictive validity of phallometrically measured sexual age and gender preference. J. Interpers. Violence 8, 486–501. (doi:10.1177/088626093008004004)

[27] Redouté J et al. 2000 Brain processing of visual sexual stimuli in human males. Hum. Brain Mapp. 11, 162–177. (doi:10.1002/1097-0193(200011)) 11098795 10.1002/1097-0193(200011)11:3<162::AID-HBM30>3.0.CO;2-A PMC6871964

[28] Zuckerman M 1971 Physiological measures of sexual arousal in the human. Psychol. Bull. 75, 297–329. (doi:10.1037/h0030923) 4931713 10.1037/h0030923

[29] Chivers M 2017 The specificity of women's sexual response and its relationship with sexual orientations: a review and ten hypotheses. Arch. Sex. Behav. Advance Online Publication (doi:10.1007/s10508-016-0897-x) 10.1007/s10508-016-0897-x 28074394

[30] Vanderah T, Gould D 2015 Nolte's The human brain: an introduction to its functional anatomy. Philadelphia, PA: Elsevier Health Sciences.

[31] Attard-Johnson J, Bindemann M 2017 Sex-specific but not sexually explicit: pupillary responses to dressed and naked adults. Dryad Digital Repository. (http://dx.doi.org/10.5061/dryad.22242) 10.1098/rsos.160963 PMC5451792 28572991

