# Supplementary material for: Sex-specific but not sexually explicit: pupillary responses to dressed and naked adults
Source: R Soc Open Sci. 2017 May 3;4(5):160963. doi: 10.1098/rsos.160963 (PMC5451792; doi:10.1098/rsos.160963)
Supplement: Fixation Data [file rsos160963supp1.docx]

**SUPPLEMENTARY MATERIALS – FIXATION DATA**

**Data Preparation**

To analyse the eye tracking data, eye movements were first pre-processed by combining fixations of less that 80ms with the preceding or following fixations if it fell within half a degree of visual angle (for similar approaches, see, e.g., Attard & Bindemann, 2014; Bindemann, Scheepers, Ferguson, & Burton, 2010). Fixations that fell outside the dimensions of the display monitor or that were obscured by eye blinks were excluded. Three regions of interest (ROIs), which comprised the head, body and scene background, were defined and the percentage of fixations that fell on these ROIs was then calculated.

**Viewing behaviour**

Observers’ percentage fixations to the ROIs for all stimulus categories are illustrated in Figure 1.

*
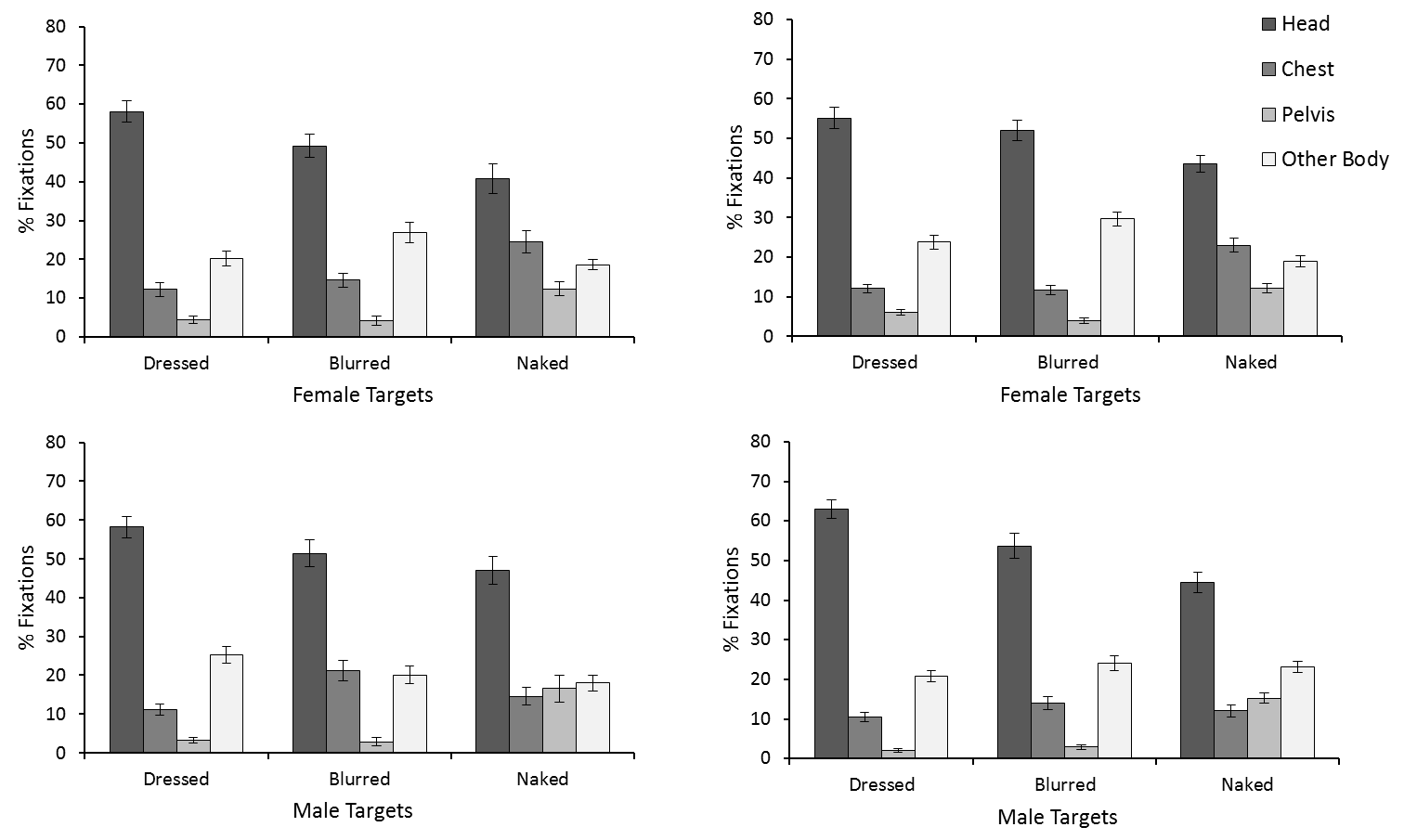
*

*Figure S1. Mean percentage fixations to the head, chest, pelvis and other body regions (arms, legs, and abdomen) for male (left) and female (right) observers. Lines represent standard errors of the means.*

Overall, 96% of fixations fell on the figures in the scenes (range = 94% to 98% across conditions), which indicates that the person-content of the scenes was of most interest. A 2 (stimulus category: men, women) x 3 (exposure: dressed, blurred, naked) x 4 (ROI: head, chest, pelvis, other body) x 2 (observer sex: male, female) mixed-factor ANOVA revealed a four-way interaction, *F*(6, 252) = 2.48, *p* < 0.05, partial η² = 0.06. To explore this interaction, a 2 (stimulus category) x 3 (exposure) x 4 (ROI) within-subjects ANOVAs was performed separately for male and female observers.

*Heterosexual Male Observers*

For male observers, this analysis showed main effects of ROI, *F*(3, 54) = 91.64, *p* < 0.001, partial η² = 0.84, but not for exposure, *F*(2, 36) = 0.64, *p* = 0.53, partial η² = 0.03, and stimulus category, *F*(1, 18) = 0.62, *p* = 0.44, partial η² = 0.03. This analysis also revealed an interaction between exposure and ROI, *F*(6, 108) = 17.85, *p* < 0.001, partial η² = 0.50. A three-way interaction was also found, *F*(6, 108) = 4.45, *p* < 0.001, partial η² = 0.20. No other interactions were revealed, all *F*s ≤ 1.59, all *p*s ≥ 0.20, partial η²s ≤ 0.08.

To explore the three-way interaction, Bonferroni-adjusted pairwise comparisons of the stimulus categories were conducted for each ROI. For female targets, more fixations were directed at the head region in dressed (57%) compared to blurred images (48%), which were both greater than the naked condition (39%), all *p*s < 0.05. Fixations for the chest in dressed (12%) and blurred images (15%) did not differ from each other, *p* > 0.74, but both showed fewer fixations to this region than nakeds (25%), both *p*s < 0.01. Percentage fixations to the pelvis did not differ for dressed (4%) and blurred images (4%), *p* = 0.10, but were greater for naked images (13%), both *p*s < 0.001. Fixations for other body regions in naked (19%) and dressed images (21%) did not differ, *p* = 0.85. Blurred (27%) stimuli elicited more fixations to the body regions than naked stimuli, *p* < 0.05, and did not differ from blurred stimuli, *p* = 0.08.

For male targets, the head was fixated on more in dressed (58%) compared to naked (47%) images, *p* < 0.05, and both did not differ from fixations to the head in the blurred condition (51%), both *ps ≥* 0.07. More fixations were directed at the chest in the blurred images (20%) compared to the dressed (11%), *p* < 0.001, but not naked images (15%), *p* < 0.05, but the latter conditions did not differ, *p* = 0.44. A greater number of fixations were directed at the pelvic region in the naked condition (17%), compared to the dressed (3%) and blurred images (3%), both *p*s < 0.01. Fixations for this region in the dressed and blurred images did not differ, *p* = 1.00. More fixations were directed at other body regions in the dressed condition (26%) compared to the naked (18%) images, *p* < 0.01, but not the blurred (21%), *p* = 0.36. Fixations for other body regions did not differ for the blurred and naked conditions, *p* = 0.51.

In summary, male observers directed more fixations to the face regions in the dressed and blurred condition, whereas when naked targets were viewed, attention was directed away from the face and shifted onto the chest and pelvic regions.

*Heterosexual Female Observers*

The equivalent analysis for female observers showed main effects of stimulus category, *F*(1, 24) =17.5, *p* < 0.001, partial η² = 0.42, and ROI, *F*(3, 72) = 95.75, *p* < 0.001, partial η² = 0.80, but not for exposure, *F*(2, 48) = 0.58, *p* = 0.56, partial η² = 0.02. This analysis also revealed interactions between exposure and ROI, *F*(6, 144) = 17.75, *p* < 0.001, partial η² = 0.43, and stimulus category and ROI, *F*(3, 72) = 4.74, *p* = 0.005, partial η² = 0.17, but not for exposure and stimulus category, *F*(2, 48) = 2.66, *p* = 0.08, partial η² = 0.10. An interaction between all three factors was also found, *F*(6, 144) = 7.11, *p* < 0.001, partial η² = 0.23.

To explore the three-way interaction, Bonferroni-adjusted pairwise comparisons of the stimulus categories were conducted for each ROI. For female targets, fewer fixations were directed at the head region in naked images (44%) compared to dressed (55%) and blurred images (both 52%), both *p*s < 0.05. Fixations for the chest in dressed and blurred images (both 12%) did not differ from each other, *p* = 1.00, but were both less than fixations to this region for naked targets (23%), both *p*s < 0.001. Percentage fixations to the pelvis was greater for naked (12%) compared to dressed images (6%), and blurred targets (4%), all *p*s < 0.01, the latter categories did not differ, *p* = 0.26. Fixations directed at other body regions did not differ for dressed (24%) and naked targets (19%), *p* = 0.37, and the latter was fewer than blurred targets (28%), *p* = 0.09.

For male targets, the head was fixated on more in dressed (63%) compared to blurred (54%) images, and both were greater than for naked images (44%), all *p*s ≤ 0.01. Fixations to the chest region did not differ for the dressed (11%), blurred (14%) and naked targets (12%), all *p*s *≥*  0.16. A greater number of fixations were directed at the pelvic region when viewing naked males (15%) compared to blurred (3%) and dressed (3%) males, both *p*s < 0.001. Fixations to the pelvic regions for the latter two conditions did not differ, *p* = 1.00. No differences were found for other body regions, all *p*s ≥ 0.36.

Overall, fixations to the face region were greater when viewing dressed and blurred targets, in contrast when viewing naked stimuli attention shifted towards the chest and pelvic region.
